# Beauty Matters: Social Preferences in a Three-Person Ultimatum Game

**DOI:** 10.1371/journal.pone.0125806

**Published:** 2015-05-07

**Authors:** Qingguo Ma, Yue Hu

**Affiliations:** 1 School of Management, Zhejiang University, Hangzhou, China; 2 Neuromanagement Lab, Zhejiang University, Hangzhou, China; University of Udine, ITALY

## Abstract

Preference for beauty is human nature, as previous behavior studies have supported the notion of “beauty premium” in which attractive people were more easily to get promoted and receive higher salaries. In the present study, 29 males were recruited to participate in a three-person ultimatum game (UG) including a proposer, a responder and a powerless third player. Each subject, playing as the responder, had to decide whether to accept an offer from the allocator both for himself and a female third person. We aimed to elucidate how the facial attractiveness of the female subject affected the male subjects’ fairness and decision-making in social exchanges. Frontal feedback-related negativity (FRN) in response to four offers in an attractive-face condition revealed no significant differences between offers; however, when the companion was an unattractive female, an “unfair/fair” offer, which assigned a lower share to the responder and a fair share to the third player, elicited the largest FRN. Furthermore, when the third player was offered the smallest amount (“fair/unfair” offer), a larger FRN was generated in an attractive-face condition than unattractive-face condition. In the “unfair/fair” offer condition in which subjects received a smaller allocation than the third person, the beauty of their female counterparts attenuated subjects’ aversion to inequality, resulting in a less negative FRN in the frontal region and an increased acceptance ratio. However, the influence of the third player’s facial attractiveness only affected the early evaluation stage: late P300 was found to be immune to the “beauty premium”. Under the two face conditions, P300 was smallest following an “unfair/fair” offer, whereas the amplitudes in the other three offer conditions exhibited no significant differences. In addition, the differentiated neural features of processing facial attractiveness were also determined and indexed by four event-related potentials (ERP) components: N170, frontal N1, N2 and late positive potentials (LPPs).

## Introduction

Social exchanges are not merely matters of goods and money; other nonmaterial factors, such as love, affection, esteem and social relationships, could be involved in economic activity. Correlations of earnings with physical appearance have been examined in multiple studies that have found that attractive persons are more likely to be hired and promoted and to earn higher salaries. The concepts of the “beauty premium” and the “plainness penalty” originated from Hamermesh and Biddle [1], who found that attractive persons received higher salaries and that plain-looking persons typically earned less than individuals with average looks. A prior experiment [2] revealed that individual decisions in the ultimatum game (UG) were influenced by the appearance of others with whom they bargained: subjects offered more to attractive individuals, even if those individuals did not demand it. However, subjects also demanded more from attractive persons, which suggested that the premium might also involve liability. A trust game conducted by Wilson and Eckel [3] introduced the concept of the “beauty penalty” attached to attractive trusters who, when they failed to meet the expectation of trustees, received greater punishment than trusters who were less attractive. Li [4] complemented this theory by applying a third-party punishment experiment and found that the “beauty penalty” only appeared between same-sex counterparts. Therefore, in a two-person UG, responders’ expectation towards proposers, expecting them to be more generous or less generous, along with responders’ preference towards proposers’ appearance influenced their final choices on whether to accept the offer.

Furthermore, the two-person game is not always reflective of reality; social exchanges occur in multiple dimensions. In a three-person ultimatum game [5], a powerless third person, who had no power to split or make decisions, was added to an asset distribution, and nothing was expected from her/him. In this type of setting, the other two roles remained proposer and responder; one proposed how to divide money, and one with veto power decided not only for himself but also for the third player. By applying electrophysiological methods, the author found that early neuron response was not modulated by the existence of the third, anonymous person, and the proposals that assigned the smallest amount of money to subjects generated the most prominent negativities in the frontal brain regions, regardless of the amount assigned to the third player. In the following study [6], individuals were invited to participate in gender-matched pairs, and one played as a responder while the other played as the third powerless person. Each pair was not acquainted before being introduced to his partner in the experiment, but the conversation was restricted to things unrelated to the experiment and once the experiment began, the two sat opposite each other without making eye contact. Electrophysiological evidence indicated that the fairness consideration of subjects with veto-power was influenced by pre-communication with the third player, and this modulation was reflected in frontal negativity in the early processing stage that appeared most negative following proposals comprising the lowest share for both recipients.

This early negativity component, which is also called feedback-related negativity (FRN), is an event-related potential thought to originate in the dorsal anterior cingulate cortex (dACC). According to reinforcement learning theory, the subjective value of a forthcoming reward is coded in the midbrain dopaminergic system, causing a phasic activation of dopaminergic neurons. Negative prediction errors induced by unfavorable outcomes initiate phasic decreases in dopamine inputs and therefore give rise to increased ACC activities, which reflect a more negative FRN amplitude [7–10]. Thus, events such as negative feedback relative to positive feedback [11–12], a loss of money compared to a gain [7, 13–14], and outcomes that deviated from formal expectations [15–18] all resulted in a larger FRN component that peaked at approximately 250ms after the onset of the event. Furthermore, unfair offers in a two-person UG, which affected the responder’s expectations and violated the social norms of equality, were also accompanied by a larger FRN in frontal brain regions [9, 19–20]. Related studies indicated that concerns for fairness could be modulated by several social factors, including social distance [21–22], social status [23–24], initial ownership [25] and social exclusion [26], which all contributed to differentiated FRN amplitudes.

ACC was also related to empathic resonance [27–30] and a sense of envy towards superior others [31]. Previous studies [32–36] had reported a correlation between empathic response and FRN generation. Participants in Fukushima and Hiraki’s study [33] were asked to observe the performance of either a human friend or computers, and a FRN-like pattern was significantly elicited only when subjects observed the outcomes of decisions made by human agents. Furthermore, self-reported measures of empathy were positively associated with the magnitude of the observational FRN, suggesting that FRN reflected a social-emotional state generated by personal empathetic responses. In a paired experiment [34], when subjects simply sat and observed gambling game with no chance to earn or lose money, the differentiated feedback-related negativity (d-FRN) was found to be much larger when they observed their friends’ loss and gain compared to those of strangers, indicating that subjects exhibited greater motivational relevance towards their friends than towards strangers. However, when subjects themselves also participated in this gambling task together with one friend and one stranger, the distinction of d-FRN between friends and stranger disappeared. A recent experiment [35] further supported the notion that in a social competition context, self-interest was dominant, and when participants received a loss due to friends’ gain a larger FRN was generated. As mentioned previously [5–6], in a three-person UG, subjects were assigned to an advantageous or a disadvantageous condition compared to the third companion. By applying a modified three-person UG, we intended to investigate how the facial attractiveness of the third player would modulate the responders’ consideration of fairness in this social comparison and determine the amplitude of the FRN.

The social comparison of payoffs was also found to affect the late processing stage, indexed by a late positive potential (LPP) [37–38]. This late positivity, P300, peaking in the period of 300-600ms in the central-to-parietal brain area, is thought to reflect attentional allocation and motivational salience [39, 40]. It has been reported to be sensitive to both the magnitude of a reward, with a more positive P300 in response to a larger payoff [8, 41–42], and the valence of a reward, with a larger amplitude for a positive outcome than for a negative one [43–45]. In an experimental task that involved asset division, a more favorable split generated a larger P300 than an unfavorable one [21, 25–26]. Additionally, an egocentric salience for the self was found to be associated with P300 in empathic responses, with a larger P300 for one’s own financial outcome than for that of one’s counterparts, regardless of the social context [36]. Although no P300 effect has been discussed in formal three-person UG papers, we might expect to uncover this effect in a facial-dependent context.

Previous behavior experiments [46–47] showed that men identified physical attractiveness as a necessity in mate selection while status and resources were valued more by women. An fMRI study [48] found that mOFC, the brain area found to reflect subjective evaluation of rewarding events, was only recruited by males to distinguish attractive and unattractive faces. Other ERP data [49] supported this notion by showing that men paid more attention to cues such as facial beauty, indexed by increased late slow wave activity for attractive faces. Based on sex differences in processing preference towards the opposite sex, we only recruited male subjects to study whether and how facial attractiveness of the female third player in a three-person UG would influence their consideration of fairness.

Additionally, we analyzed subjects’ ERPs during face presentation to measure whether they could distinguish between attractive and unattractive faces. Although brain-imaging studies have shown increased activity in the reward-related brain area when subjects perceive attractive faces compared to unattractive faces [50–55], ERP with a higher time resolution is able to reveal a more precise processing time course. Werheid [55] reported enhanced LPP amplitude in response to attractive relative to unattractive faces, which was consistent with previous findings [56–57]. Meanwhile, a greater posterior negativity was observed as soon as 250ms after stimuli, which suggests that the appraisal of facial attractiveness begins during an earlier stage of processing [55]. The same two temporal stages of processing were reported for attractive and unattractive faces [58] but yielded contrary N2 and LPP patterns. Van Hooff [59] examined the attention bias towards attractive opposite-sex faces and observed a similar increased slow wave effect that indexed enhanced evaluative processing and motivated attention in male participants. Given the various findings and controversies in the literature, we wished to explore the related ERP components and provide neural markers to show that subjects really considered attractive and unattractive faces as such.

## Materials and Methods

### Participants

Twenty-nine right-handed male subjects participated in this study. They were all students at Zhejiang University and aged 18–27 years (M = 23.56 years, SD = 2.45years). They were all native Chinese speakers, had normal or corrected-to-normal vision, and did not have any history of neurological disorders or mental disease. All participants provided written informed consent to participate in this study, and the study was approved by the Neuromanagement Laboratory ethics committee at Zhejiang University. Data from three subjects were discarded, one for excessive recording artifacts and two for misunderstanding the rules of the game, resulting in twenty-six valid subjects for the final data analysis.

### Stimulus material

The stimuli included 256 photos of Chinese females that were collected from the CAS-PEAL-R1 Face Database, photo pools of PKU and CAS and the Internet. The faces were unfamiliar to subjects and included no movie stars, singers or other celebrities. All faces were grey-processed using Photoshop software to ensure consistency in background, brightness, contrast, and color saturation and adjusted to a uniform size (4.5 by 4 cm, 220 by 200 pixels). All images were rated for attractiveness (from 1 = 'not attractive at all’ to 7 = ‘extremely attractive’) by 20 male students. The ratings of the two categories of faces were compared by a paired t-test, wherein the attractiveness was significantly different (M_attractive_ = 5.2, M_unattractive_ = 2.11; t (19) = 14.516, p<0.001).

Four varied offers were presented during the game, representing different divisions of the amount of ¥12 between three players: “fair/fair” offer (each received ¥4), “unfair/unfair” offer (¥10 for the allocator, ¥1 each for the responder and the third player), “fair/unfair” offer (¥7 for the allocator, ¥4 for the responder, ¥1 for the third player) and “unfair/fair” offer (¥7 for the allocator, ¥1 for the responder, ¥4 for the third player). In total, 256 offers and 256 photos were paired and distributed evenly in four blocks with eight conditions, 2 (face: attractive and unattractive) × 4 (offer: “fair/fair”, “unfair/unfair”, “fair/unfair” and “unfair/fair”), each containing 32 trials.

### Experimental procedure

Subjects were seated comfortably in a dim, sound-attenuated and electrically shielded room. The experiment was introduced on paper handouts. The experimental stimuli were presented at the center of a computer screen at a distance of 100 cm from each subject’s face. A keypad was provided to the subjects to make their choices. Before the experiment formally began, each subject had 20 practice trials to become familiar with the experimental procedure.

**Fig 1:**
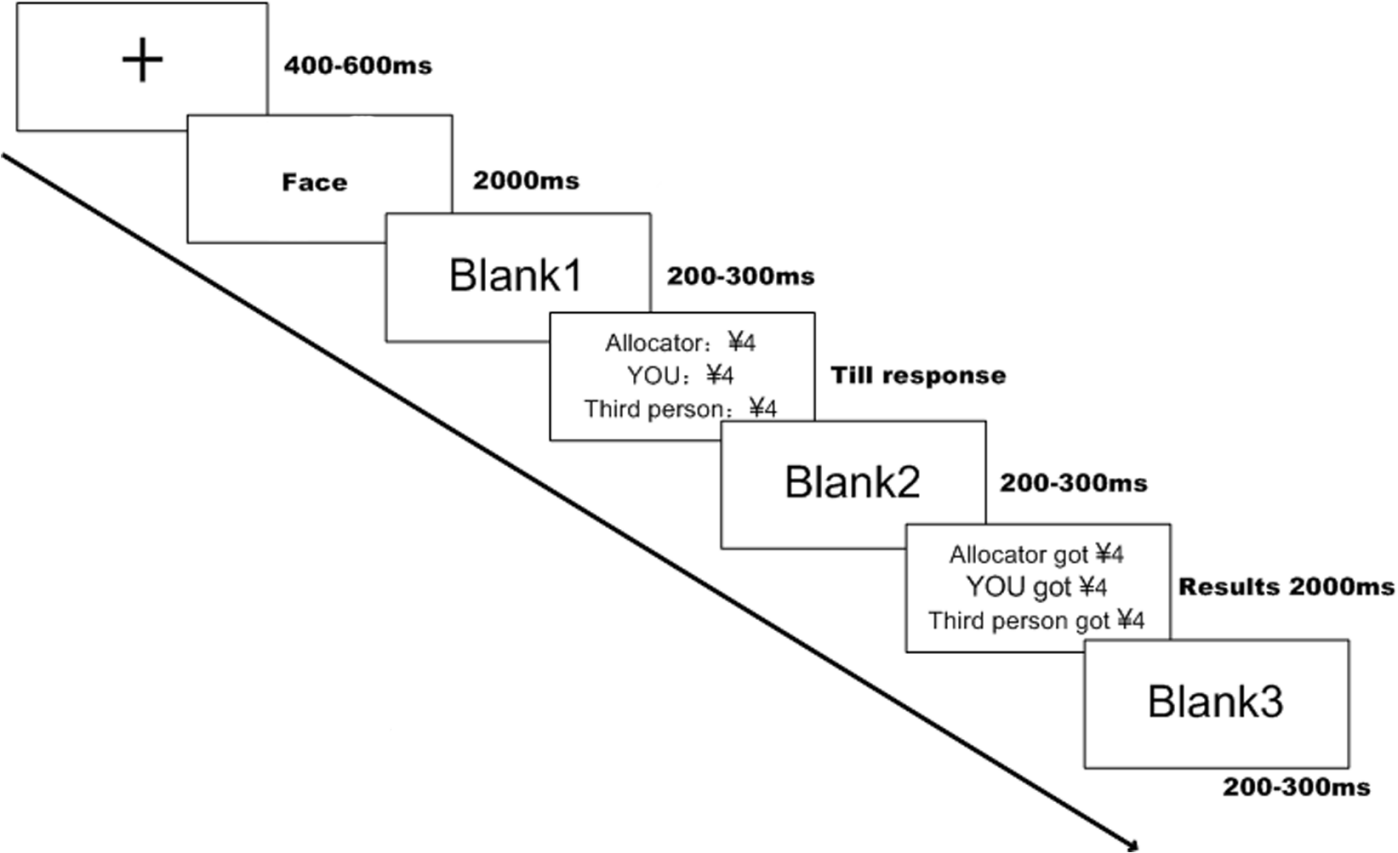
A single trial of the experimental procedure. Participants first saw either an attractive face or an unattractive face before the presentation of the offers. They made their choice by pressing the keypad and had unlimited time to make a decision. Screen then showed the final payoffs.

A single trial is illustrated in Fig 1. A fixation appeared at the beginning of each trial for 400-600ms on the black screen. Then, a photo of the third player was presented for 2000ms, followed by the allocator’s proposal of how to split ¥12 among the three players, written in Chinese. Subjects were informed that the proposals were collected in a prior behavior experiment, and each subject, as the responder, had to decide whether to accept the offer by pressing the keypad after thoroughly thinking not only for himself but also for the powerless third player, whose real payoff depended on subjects’ decisions that each recruited female player would receive the exact amount of money she was allocated after the experiment. If the subject chose to accept the offer, then he and the female third player would receive the amount of money the proposer suggested; otherwise, all of them would receive nothing. Once he made his decision, the final payment of that trial was presented on the screen for 2000ms before continuing to the next trial. The payment for their participation was ¥30 (approximately $4.80) plus the income from two randomly selected trials. Counterbalance was manipulated among the subjects that half of the subjects were required to press “1” for accepting and “3” for declining while the other half received opposite instructions. The E-prime 2.0 software package (Psychology Software tools, Pittsburgh, PA, USA) was used for stimuli presentation, triggers and response recording.

### Electrophysiological recordings

Scalp voltages were recorded (band-pass 0.05–70 Hz, sampling rate 500 Hz) with a NeuroScan SynAmps2 Amplifier (Scan 4.3.1, Neurosoft Labs, Inc. Virginia, USA), using a 64-channel electro cap with Ag/AgCl electrodes, in accordance with the standard international 10–20 system. A cephalic (forehead) location was connected to the ground. The left mastoid was chosen for reference, and recorded EEGs were off-line re-referenced to the average of the left and right mastoids. For eye movement artifact correction, an electro-oculogram (EOG) was recorded from electrodes placed 10 mm from the lateral canthi of both eyes (horizontal EOG) and above and below the left eye (vertical EOG). The electrode impedance was maintained below 5 kΩ during the recording.

### Data analysis

To analyze the behavioral data, repeated measures ANOVA was adopted to compare the acceptance ratio and reaction time across eight conditions, 2 (face: attractive and unattractive) × 4 (offer: “fair/fair”, “unfair/unfair”, “fair/unfair” and “unfair/fair”). *Post-hoc* analysis was conducted using the Bonferroni correction.

EEG data were analyzed using NeuroScan 4.3.1. EOG artifacts were corrected first, followed by digital filtering through a zero phase shift (low pass at 30 Hz, 24 dB/octave). The EEGs were segmented for 1000ms in each epoch, beginning 200ms before and continuing until 800ms after the onset of both the face and offer presentations. The entire epoch was then baseline-corrected by the 200ms interval prior to the stimulus onset. Trials that contained amplifier clipping, bursts of electromyography activity, or peak-to-peak deflection exceeding ±80 μV were excluded from the final average. To further improve the quality of the data, subjects with fewer than 15 trials were excluded, resulting in an average of 26 trials for each condition.

During the face presentation, the EEG epochs were averaged for attractive and unattractive faces. During the offer presentation, the EEG epochs were separately averaged for face (attractive/unattractive face) × valence (“fair/fair”, “unfair/unfair”, “fair/unfair”, and “unfair/fair”), resulting in eight conditions in total. ERPLAB toolbox 4.0.2.3 was used to generate topographic maps for each condition [60].

Based on the visual observation of grand average waveforms and previous ERP studies on outcome processing [5–6, 26], two ERP components—frontal FRN and parietal P300—were analyzed. We averaged the ERP amplitude of the time ranges of 290-370ms and 400-600ms post-onset of the offer presentations for FRN and P300 analysis, respectively. We selected six electrode sites—F1, Fz, F2, FC1, FCz and FC2—in the frontal-central areas for FRN analysis and nine electrode sites—C1, Cz, C2, CP1, CPz, CP2, P1, Pz and P2—in the central and parietal areas for P300 analysis. The amplitude values of the FRN were submitted to 2 × 4 × 6 repeated measures ANOVAs with the factors face (attractive and unattractive), valence (“fair/fair”, “unfair/unfair”, “fair/unfair” and “unfair/fair”) and electrodes (F1, Fz, F2, FC1, FCz and FC1). A similar analysis was performed for P300 with nine electrodes (C1, Cz, C2, CP1, CPz, CP2, P1, Pz and P2) included. The Bonferroni correction and the Greenhouse-Geisser correction were applied in all statistical analyses when necessary. A simple effect analysis was conducted when the interaction effect was significant.

To examine the neural processing of facial attractiveness, the mean amplitude of N170 (P7, PO7, P8 and PO8), frontal N1 (F1, FZ, F2, FC1, FCZ and FC2), frontal N2 (F1, FZ, F2, FC1, FCZ and FC2) and central-parietal LPP (C1, Cz, C2, CP1, CPZ, CP2, P1, Pz and P2) were further analyzed for the ranges of 150-180ms, 110-130ms, 280-360ms and 450-650ms post-onset of face presentation, respectively. Two-way ANOVAs were conducted on the N2 and LPP components. The ANOVA factors were face (attractive and unattractive) and electrode sites. The degree of freedom of the F-ratio was corrected according to the Greenhouse-Geisser method.

## Results

### 3.1 Acceptance ratios

**Fig 2:**
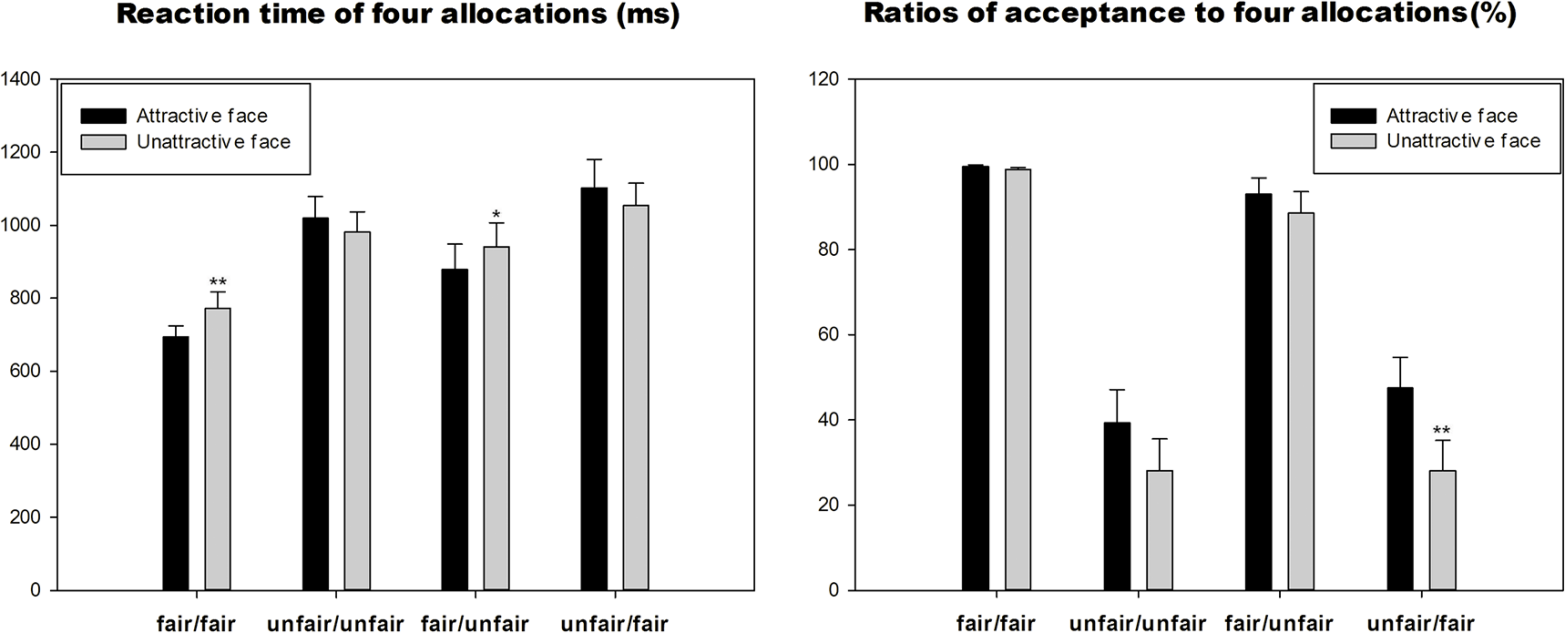
The reaction time and acceptance ratio of four offers in two face conditions. (*p<0.05; **p<0.01).

The ANOVA analysis for the acceptance ratio (Fig 2) revealed main effects of face (F_1, 25_ = 13.021, *p* = 0.001) and offer (F_1, 25_ = 54.383, *p*<0.001). Generally, the acceptance ratio in the attractive-face condition was higher (*p* = 0.001) than that in the unattractive-face condition, as with the post-hoc comparisons between the two fair offers (“fair/fair” and “fair/unfair”) and two unfair offers (“unfair/unfair” and “unfair/fair”) assigned to subjects, with all of the four significant levels were below 0.001. The comparison between the “fair/fair” and “fair/unfair” offers was not significant (*p* = 0.368), and neither was the comparison between the “unfair/unfair” and “unfair/fair” offer conditions (*p* = 1.000), neither was the comparison between “unfair/unfair” and “unfair/fair” offers (*p* = 0.694).

The interaction effect also revealed a significant difference (F_1, 25_ = 4.659, *p* = 0.012), and the post-hoc comparison demonstrated that the acceptance ratio of the “unfair/fair” offer in the attractive-face condition (mean±SE, 47.5±7.2%) was higher than in the unattractive-face condition (mean±SE, 28±7.2%), *p* = 0.001. The comparisons between acceptance ratios of “unfair/unfair” offer in two face conditions was marginally significant (*p* = 0.065). Between the two face conditions, no other apparent differences were found regarding the acceptance ratios of “fair/fair” (*p* = 0.134) and the “fair/unfair” (*p* = 0.109) offers.

### 3.2 Response time

The ANOVA analysis of the response time (Fig 2) revealed a main effect of offer (F_1, 25_ = 15.429, *p*<0.001). The main effect of face was not significant (F_1, 25_ = 0.925, *p* = 0.345). Post-hoc comparison showed that subjects responded quickest to the “fair/fair” compared to the “unfair/unfair” (*p*<0.001), “fair/unfair” offer (*p* = 0.001) and “unfair/fair” offer (*p*<0.001). The response time to the “fair/unfair” offer condition was not significantly different from those to the “unfair/unfair” (*p* = 1.000) and “unfair/fair” (*p* = 0.072) conditions.

The interaction effect revealed a significant difference (F_1, 25_ = 4.69, *p* = 0.005): The reaction time of the “fair/fair” offer was shorter (*p* = 0.009) in the attractive-face condition (mean±SE, 694.066±29.97ms) than in the unattractive-face condition (mean±SE, 771.49±45.60ms). While in the “fair/unfair” offer condition, subjects responded more rapidly in the attractive face-condition than in the unattractive-face condition (*p* = 0.04). No significant difference was found between two face conditions in the “unfair/unfair” (*p* = 0.122) and “unfair/fair” offers (*p* = 0.199). The acceptance ratio and reaction time of each condition are reported in S1 Fig

### 3.3 FRN

**Fig 3:**
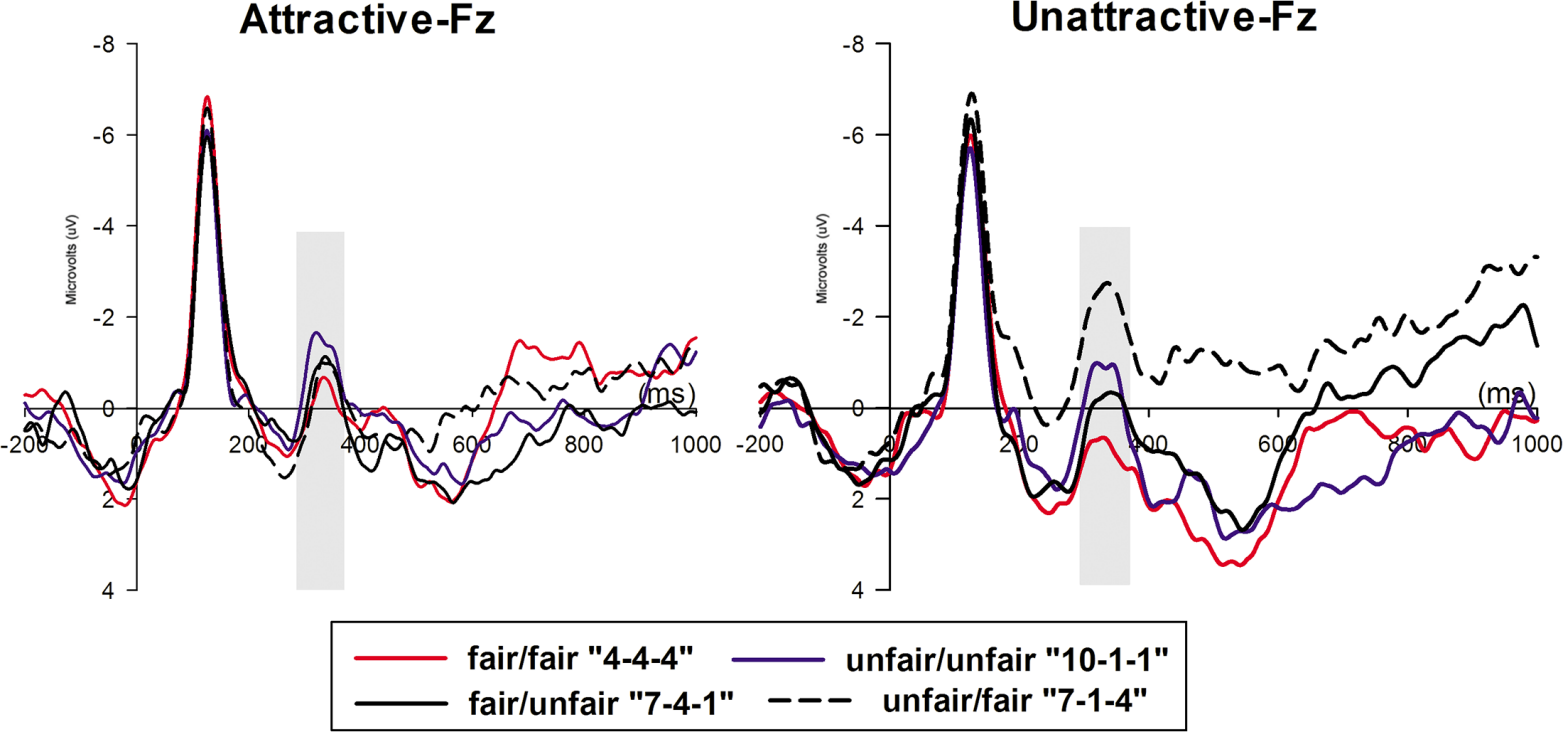
The ERP grand-average waveforms of FRN at Fz in two face conditions. The time window for FRN was 290-370ms.

The ERP grand average waveforms at channel Fz during the presentation of an offer are depicted in Fig 3. The ANOVA analysis of the FRN revealed a main effect of offer (F_1, 25_ = 9.038, *p*<0.001) and electrode (F_1, 25_ = 16.372, *p*<0.001) but no main effect of face (F_1, 25_ = 0.708, *p* = 0.408). The “fair/fair” offer generated the smallest FRN, and post-hoc comparisons revealed a marginally significant difference from the “unfair/unfair” offer (*p* = 0.053), and a significant difference compared with the “unfair/fair” offer (*p* = 0.001). Additionally, the amplitude of the FRN in response to the “fair/unfair” offer was more negative-going than the FRN for the “unfair/fair” offer (*p* = 0.008). However, this contrast did not produce a significant effect (*p* = 0.318) for the comparison between the “fair/unfair” and “unfair/unfair” offers.

Moreover, the interaction effect between face and offer was significant (F_1, 25_ = 8.83, *p*<0.001), while the interaction effect among face, offer and electrode was not significant (F_1, 25_ = 1.079, *p* = 0.374). In the attractive-face condition, FRNs in response to four offers revealed no significant differences from one another. In the unattractive-face condition, the largest FRN was in response to “unfair/fair” offer, which revealed a remarkable difference from the other three offers (all three *p*-values were below0.001). Additionally, in the unattractive-face condition, the FRN generated in response to the “unfair/unfair” offer was more negative than the FRN in response to the “fair/fair” (*p* = 0.006) and “fair/unfair” (*p*<0.001) offers. No other significant comparisons were found between the offers in the unattractive-face condition. Waveforms of FRN at Fz regarding the four offers in each face condition are presented in Fig 3; FRN peaked highest at this site (mean±SE, -1.440±0.517μV).

**Fig 4:**
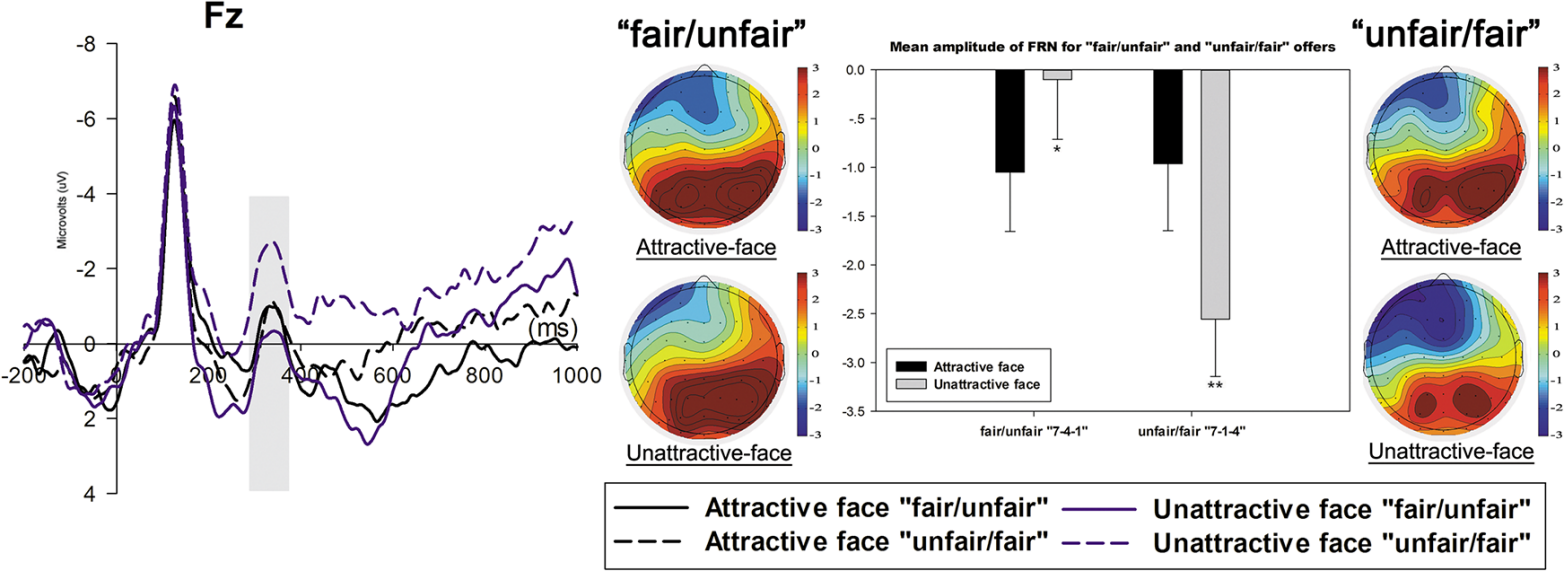
The comparison of the grand-average waveforms of FRN for the “unfair/fair” and “fair/unfair” offers at Fz and topographical maps. The time window for FRN was 290-370ms. The four maps on the right illustrate the topographical distribution of FRN with the time window “290-370ms” in the corresponding face condition. The bar chart depicts the comparison between the mean amplitude of the FRN for the “fair/unfair” and “unfair/fair” offers in two face conditions. The bar for the topographical map ranges from +3μv to -3μv. (*p<0.05; **p<0.01).

Additionally, the FRN was more negative (*p* = 0.047) in response to the “fair/unfair” offer in the attractive-face condition than in the unattractive-face condition. In response to “unfair/fair” offer, the comparison between the amplitudes of the two FRNs in two face conditions showed a reversed pattern in which the FRN was more positive (*p* = 0.001) in the attractive-face condition than in the unattractive-face condition (Fig 4). The FRN amplitude of each condition is reported in S1 Fig Regarding the “unfair/unfair” (*p* = 0.182) offers, no significant differences was found between the two face conditions, while comparisons for “fair/fair” offer approached significance (*p* = 0.064).

### 3.4 P300

**Fig 5:**
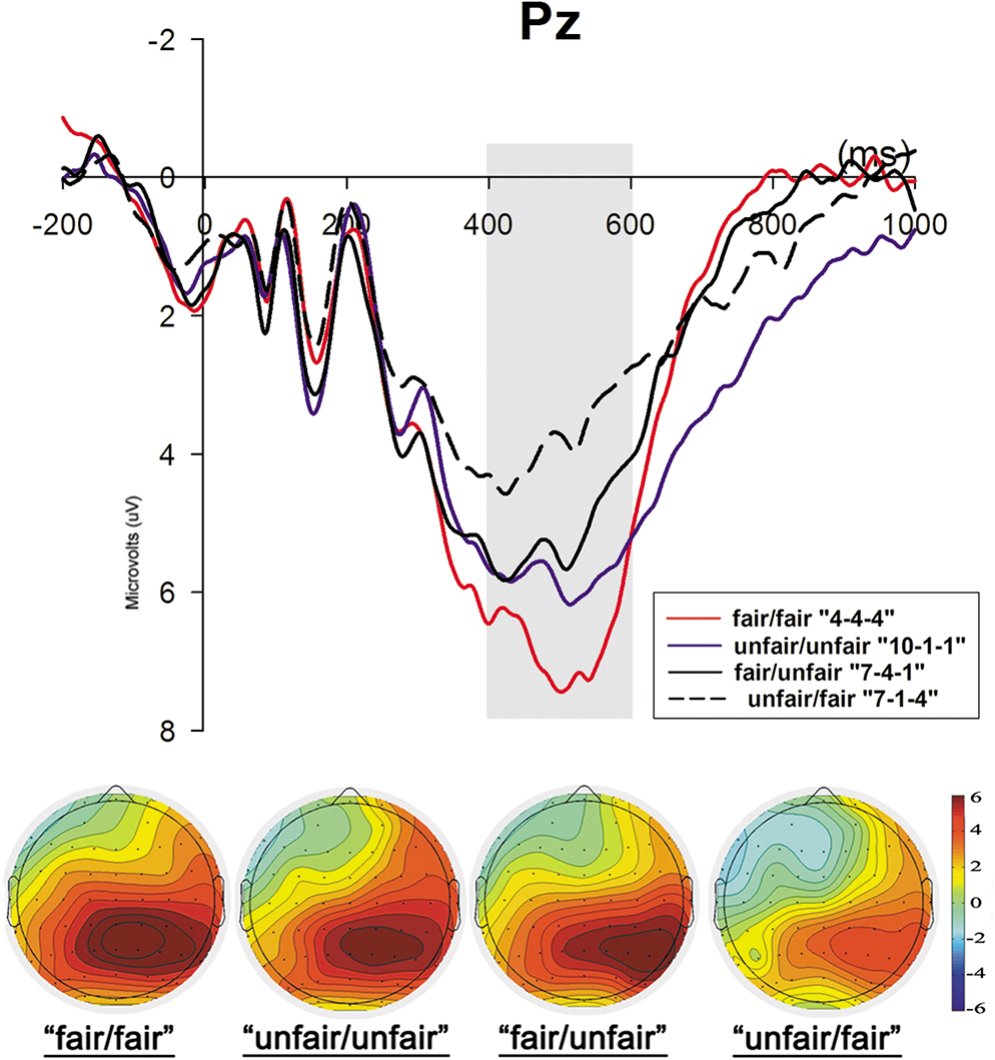
The ERP grand-average waveforms of P300 for four offers in the two face conditions at Pz and topographical maps for the four offer conditions. The time window for P300 was 400-600ms and the bar for the topographical map ranges from +6μv to -6μv.

Regarding the central-parietal component P300 (Fig 5), we observed a significant main effect for offer (F_1, 25_ = 13.834, *p*<0.001) and electrode (F_1, 25_ = 24.280, *p*<0.001), but no main effect of face (F_1, 25_ = 3.342, p = 0.079). Post-hoc comparisons indicated that the P300 amplitude was highest in the Pz electrode (mean±SE, 5.423±0.776μV), and P300 for the “unfair/fair” offers was smaller than P300 for other three offers, i.e., the “fair/fair” (*p*<0.001), “unfair/unfair” (*p*<0.001) and “fair/unfair” (p = 0.007) offers. Apart from these differences, no other significant differences were found. The interaction effect between face and offer was not significant (F_1, 25_ = 1.983, *p* = 0.124), and neither was the interaction effect among face, offer and electrode (F_1, 25_ = 0.546, *p* = 0.963), which suggested that facial attractiveness had no effect on the late processing stage. The P300 amplitudes of each condition are reported in S1 Fig

### 3.5 N170, frontal N1, N2 and LPP

**Fig 6:**
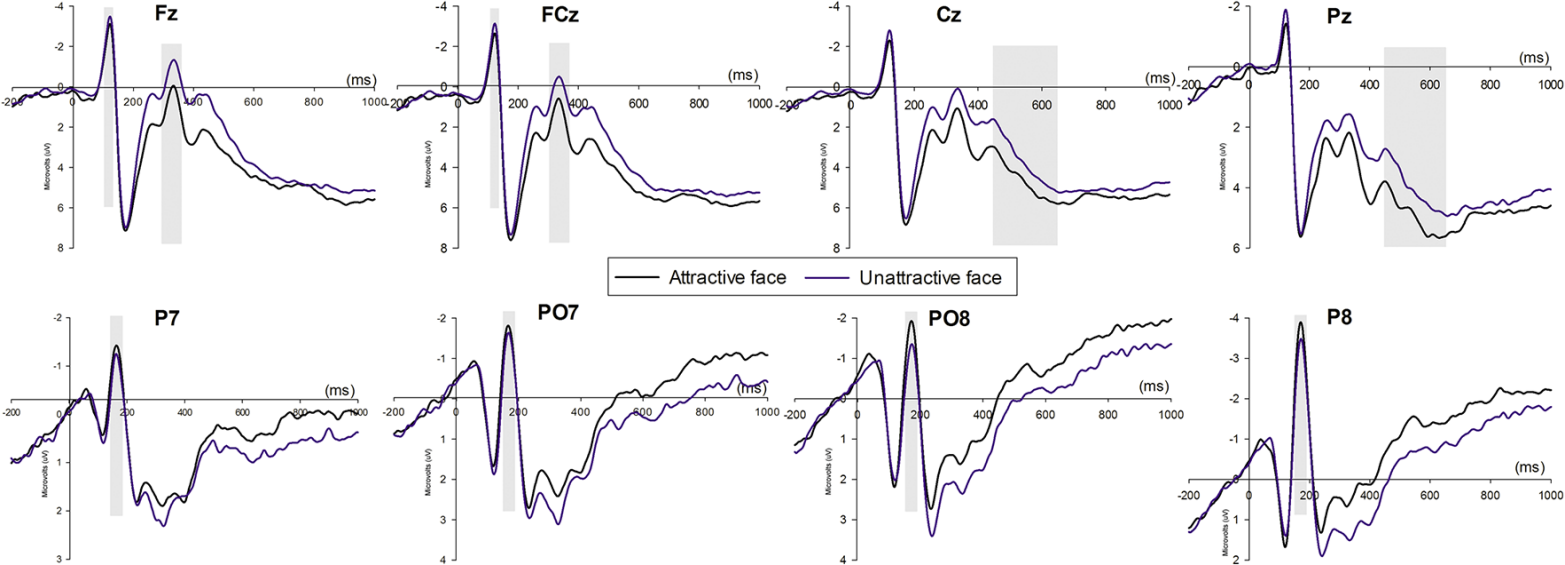
The ERP grand-average waveforms of N1, N2 and LPP at the midline Fz, FCz, Cz, and Pz, and N170 at P7, P8, PO7 and PO8 for attractive face and unattractive face conditions. The time window was 150-180ms for N170, 110-130ms for N1, 280-260ms for N2, and 450-650ms for LPP.

The ANOVA analysis for N170, frontal N1, frontal N2 and parietal LPP (Fig 6) revealed main effects of face: A more negative frontal N1 (F_1, 25_ = 5.318, *p* = 0.03) and frontal N2 (F_1, 25_ = 11.557, *p* = 0.002) were elicited by unattractive faces, and a more negative N170 (F_1, 25_ = 4.745, *p* = 0.039) and a more positive LPP (F_1, 25_ = 10.032, *p* = 0.004) was elicited by attractive faces. The main effect of electrode (F_1, 25_ = 5.569, *p* = 0.003) and the interaction effect between electrode and face (F_1, 25_ = 4.21, *p* = 0.011) were significant for frontal N2. Unattractive faces elicited a more negative N2 than attractive faces at all selected electrodes. In attractive-face condition, N2 peaked highest at the Fz electrode (mean±SE, 1.43±0.599μV) while in the unattractive-face condition, the most negative N2 was found at the FC2 electrode (mean±SE, -0.043±0.579μV).

However, for LPP, neither the main effect of electrode (F_1, 25_ = 2.575, *p* = 0.095) nor the interaction effect between electrode and face was significant (F_1, 25_ = 1.029, *p* = 0.393). For frontal N1 and N170, the main effect of electrode (F_1, 25_ = 4.26, *p* = 0.008; F_1, 25_ = 4.745, *p* = 0.039, respectively) both reached significance, while the interaction effects between electrode and face were not significant (F_1, 25_ = 2.278, *p* = 0.087; F_1, 25_ = 0.809, *p* = 0.463, respectively).

## Discussion

This study examined how the facial attractiveness of a third, passive player would influence the behavioral and neural responses of the responder in a modified ultimatum game. Behavioral results revealed the difference. The general tendency of the acceptance ratio was consistent with previous findings [9–10, 61] that participants preferred fair shares, and offers with lower shares for the responder were more likely to be rejected. More importantly, the acceptance ratio was higher in the attractive-face condition than in the unattractive-face condition. When subjects encountered a disadvantageous-unfair (“unfair/fair”) offer in which they were allocated a smaller share than the third player, they accepted more offers if the third female player was attractive, which confirmed the theory of the “beauty premium”. Additionally, male subjects reacted more rapidly to fair offers (“fair/fair” and “fair/unfair”) in the presence of an attractive female third player.

The ERP data supported the behavioral results. As we outlined previously, FRN was more negatively deflected to undesirable or unexpected outcomes [15–21]. In the attractive-face condition, no significant differences were found between FRNs in four offer conditions. However, in the unattractive-face condition, except the comparisons between two fair offers assigned to subjects (”fair/fair” and “fair/unfair”) and two unfair offers assigned to the third player (“unfair/unfair” and “fair/unfair”) revealed no significant differences, other comparisons between offer conditions all reached statistic significances. When the third player was attractive, the FRN was smaller following the “unfair/fair” offer and larger following the “fair/unfair” offer than in the unattractive face condition. Although the FRN was modulated by the facial attractiveness of the third person, the results of the P300 amplitude revealed no interaction between the facial attractiveness of the third person and the valence of the offers. In response to the “unfair/fair” offer, the P300 was smallest among the four offer conditions, and the other three conditions revealed no differences in the mean amplitude.

Fehr and Schmidt [61] advanced a theory of inequity aversion holding that individuals were more concerned with the fairness of their own payoff than with the payoffs of others. In line with this theory, subjects in previous studies [9–10] denied two-thirds of disadvantageous and unfair offers and accepted more than half of advantageous offers. Our data demonstrated that the facial attractiveness of the third player provoked altruistic behavior in responders [62] and indicated that nearly half of the smaller shares (47.5%) assigned to responders were accepted in the attractive-face condition, a remarkably higher percentage than in the unattractive-face condition (28%). Moreover, the attractiveness of the third player significantly facilitated subjects’ response process to “fair/fair” offer and also increased the acceptance ratio of equally unfair offers (“unfair/unfair”) which suggested an implicit preference for sharing a fair offer with attractive females.

FRN is thought to reflect the deviation between the actual outcome and prior expectations. When individuals experienced an undesirable outcome, more negative activity was observed in the frontal regions. Based on the grand-averaged ERPs in the present study, we found apparent differences in the FRN amplitude in different offer conditions: FRNs were larger following unfair offers than following fair offers. This difference in FRN among the four separate offers, due to a violation of social norms, was only significant in the unattractive-face condition. In the unattractive-face condition, the most pronounced FRN, which indicated the responders’ most disfavored outcome, was elicited by the “unfair/fair” offer in which responders received the smallest amount of money. However, no such difference was observed in the attractive-face condition: fair offers (“fair/fair” and “fair/unfair”) and unfair offers (“unfair/unfair” and “unfair/fair”) assigned to the subjects themselves generated similar FRN amplitudes. A similar pattern was observed in a dictator game [21] when participants played with strangers or with one of their friends. Friendship was thought to be a factor that promoted an egalitarian distribution of assets; therefore, the feelings and judgments of the strangers were weakened. In our experiment, the situation was reversed. It appeared that male responders’ dissatisfaction with comparative inequality was stronger in unattractive-face condition than in attractive-face condition. Considering the rewarding effect of beauty, we posited that it was the subjects’ preference for and enjoyment of facial attractiveness that weakened their consideration of fairness, decreased FRN responses for the comparative processes and resulted in the null effect of FRN.

In addition to the differentiated FRN between all offers assigned to the responders in the two face conditions, the FRN pattern varied following advantageous (“fair/unfair”) and disadvantageous (“unfair/fair”) offers. As outlined above, the ACC was activated when the subjects experienced a sense of envy for others who possessed superior quality and ability [31], and increased ACC activity led to increased FRN amplitude. In our study, when the responder received the smallest offer (“unfair/fair”), he might have felt economically inferior to the third player and this feeling might have elicited a larger FRN in the frontal area. Because the current data revealed that the FRN for an “unfair/fair” offer was smaller in the attractive-face condition than in the unattractive-face condition, we presumed that the attractiveness of “others” might abate responders’ feeling towards the higher share of the third players and therefore lead to a decrease in FRN amplitude. Along with the increased acceptance of the “unfair/fair” offer in the attractive-face condition, the comparison of FRN amplitudes indicated that the beauty of the female triggered prosocial traits among male subjects and enhanced their altruistic actions in this experiment.

This FRN pattern was found to be reversed when responders were in an advantageous (“fair/unfair”) condition. The ACC was also thought to code the subjects’ affective experience of empathy when perceiving the pain or distress of others [27] and FRN was generated in this brain region as mentioned previously [7–10], which suggested that subjects’ empathic response to the suffering and fortune of others was related to this early component [32–36]. Several social factors affected the modulation process of subjects’ empathic response to the errors, losses and gains of others as indexed by the amplitude of the FRN [34, 63–66]; as a social species, the humans’ preference for beauty is innate and has evolved from ancient survival to modern sociality. Perceiving attractive faces aroused the subjects’ concerns for the fair treatment of the third players and enhanced their internal empathy when the third players were treated unequally. This consideration consequently resulted in a more negative FRN for fair/unfair offers.

Another ERP component, P300, was thought to represent the motivational importance of reward [8, 67–68]. The “unfair/fair” offer, in which the responder was unequally treated and assigned the smallest share, generated the smallest P300 in the central-parietal area in the two face conditions, which is consistent with previous findings that the magnitude of P300 is less positive in response to unequal offers [25–26, 37]. However, as no interaction effect between face and offer was found to be significant, we presumed that the facial attractiveness of the third player had no effect on the late evaluation stage.

Furthermore, in agreement with an ERP result from a social comparison task [69], the amplitude of P300 with respect to the “unfair/unfair” offer was much more positive than that with respect to the “unfair/fair” offer and revealed no significant difference from P300 for the other two fair shares (“fair/fair” and “fair/unfair”). This result indicated that although responders also received ¥1 from the two unfair offers, the equally unfair (“unfair/unfair”) offer evoked higher-level motivational evaluation and attention than unequally unfair (“unfair/fair) offer in the subjects’ late neural processes. A previous UG study [37] also revealed that moderately unequal offers evoked more positive LPPs than highly unequal offers only when subjects received more than recipients in other groups, indicating that LPP was more sensitive to social comparison rather than to offer type. Although LPP might differ from P300 in scalp distributions and in time range, previous studies indicated that the two potentials shared some similar functions in stimulus evaluation and emotional processes [70]. In this three-person UG, we provided further proofs on P300’s higher sensitivity to comparison between responders and their companions than to the amount of monetary shares or the offer type. More importantly, this P300 pattern was independent of the effect of the “beauty premium” of their companions.

Notably, the pattern of the FRN effect outlined above was inconsistent with the pattern of the effect in acceptance ratios; in the attractive-face condition the “unfair/fair” offer was accepted more often and accompanied by a decreased FRN, whereas a higher FRN in response to the “fair/unfair” offer was not accompanied by a differentiated acceptance ratio. The apparent contradiction between the ERP pattern and behavior data was also reported in previous studies [9, 37, 71]. In our study, the facial attractiveness of the third player was only found to modulate the early affective responses indexed by FRN and exerted no influence on the late neural processes indexed by P300. The unlimited response time given to participants to allow them to perform an elaborative thinking and strategic evaluation might also contribute to the discrepancy between the final choices and early neuronal activities. In a UG that included social exclusion [26], all fair offers were accepted in three groups of allocators, regardless of the initial social exclusion effect. In a similar situation, subjects were mainly driven by self-interest which overshadowed all other social factors.

The ERP data also illustrated how the brain responded differently to attractive and unattractive faces. LPP was elicited to a greater extent in response to attractive faces than to unattractive faces, which was in line with previous findings [54–57, 59]. LPP was thought to reflect deliberative processing and motivational significance [70], and facial attractiveness, which was perceived as a reward and had evolutionary importance, would draw more attention than unattractive faces, therefore resulting in increased positivity in posterior areas. Furthermore, two earlier negativities, frontal N2 and N170, were both more negative in the unattractive face condition; while N170 was elicited higher in attractive-face condition. N2 was sensitive to the valence of stimuli, and negative stimuli were found to be associated with increased N2 [72–74]. In the current study, the N2 effect could be interpreted as different responses to favored and disfavored stimuli that disfavored unattractive faces and elicited a more negative N2. In addition to the early (P2 and N2) and late (LPP) potentials discussed in prior studies [55–58], we further revealed that an earlier negativity, peaking at approximately 120ms post stimuli, also responded differently to attractive and unattractive faces. The frontal N1 was found to be more negative for the faces of untrustworthy persons [75], who were associated with a higher processing demand. N1 was also shown to indicate early attention-related processing and preparation for later efficient conflict processing [76]. Thus, changes in frontal N1 could be associated with N2 and were together modulated by facial attractiveness. In addition, occipito-temporal regions was reported to involve in face categorization [77] and reflected in N170 [78], therefore the differentiated N170 found in two face conditions indicated that attractive and unattractive faces can be identified in an earlier stage.

## Conclusions

To summarize, in a three-person ultimatum game, the facial attractiveness of the third player modulated responders’ fairness in the early evaluation process, as indexed by FRN, whereas late P300 was immune to the effect of the “beauty premium” and was dominantly influenced by the comparative process. Behaviorally, increased altruism and attenuated envy towards the third players’ superior gain provoked subjects’ altruistic actions in a disadvantageous offer condition when the third player was attractive. In addition, the ERP associated with processing facial attractiveness was also examined. In addition to N2 and LPP, which were explored in previous studies, an earlier frontal N1 and N170 was found among the different neural responses to attractive and unattractive faces.

## Supporting Information

S1 Fig The amplitudes of FRN and P300, acceptance ratio and reaction time of four offers in two face conditions (mean±SE). (TIF)

S1 File Zip file containing raw experimental data. (ZIP)
