# Supplementary figures and images for: Beauty Matters: Social Preferences in a Three-Person Ultimatum Game
Source: PLoS One. 2015 May 7;10(5):e0125806. doi: 10.1371/journal.pone.0125806 (PMC4423917; doi:10.1371/journal.pone.0125806)

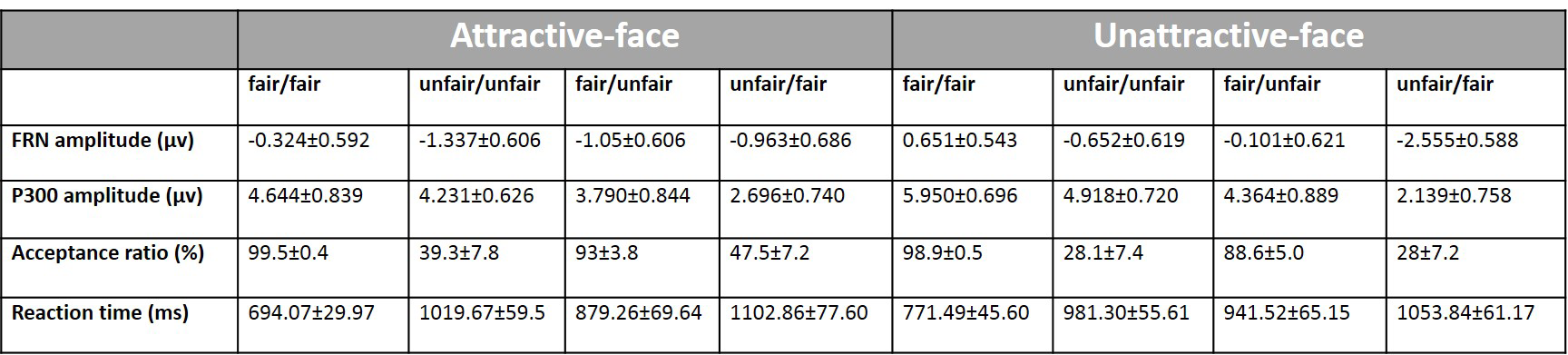

Supplement: S1 Fig — (TIF) [file pone.0125806.s001.tif]
